# Insights into amoxicillin pharmacokinetics using physiology-based pharmacokinetic modelling

**DOI:** 10.1128/aac.00465-26

**Published:** 2026-07-17

**Authors:** Christopher A. Darlow, Vineet Dubey, Nada Reza, William Hope

**Affiliations:** 1 Antimicrobial Pharmacodynamics and Therapeutics, University of Liverpool 4591, Liverpool, United Kingdom; University of Houston, Houston, Texas, USA

**Keywords:** probenecid, urinary pharmacokinetics, PK/PD, pharmacokinetics, *Haemophilus influenzae*, *Streptococcus pneumoniae*, physiology-based pharmacokinetic modeling, amoxicillin

## Abstract

Amoxicillin is the most commonly used antibiotic globally. However, there is a relatively poor understanding of its pharmacokinetics, pharmacodynamics, and clinical pharmacology. We constructed a physiology-based pharmacokinetic (PBPK) model of amoxicillin to gain deeper insights into the adequacy of amoxicillin regimens for the treatment of infections relevant to global health. We constructed an amoxicillin PBPK model in PK-Sim using known ADME and physicochemical parameters, *in vitro* characterized transporter kinetics of amoxicillin with OAT3, and time-concentration data from the published literature. Unknown parameters were fitted using a subset of available clinical pharmacokinetic data for training, before final validation with a holdout data set. Population simulations were performed using the final model for a range of amoxicillin regimens and contexts. The final amoxicillin PBPK model was high performing by fold-error metrics for both plasma and urinary concentrations. Simulations demonstrated all regimens achieved the 40%T>minimum inhibitory concentration (MIC) target for *Streptococcus pneumoniae* up to 1 mg/L and all except p.o. 500 mg amoxicillin q8h for the wild-type *Haemophilus influenzae* MIC distribution. The simulations also demonstrated high urinary amoxicillin exposures and evaluated the effects of inadequate active ingredient content, missed doses, and probenecid co-administration. This PBPK model gives the following insights into amoxicillin pharmacokinetics: i) the adequacy of amoxicillin for infections caused by *S. pneumoniae* and *H. influenzae*; ii) the impact of non-compliance and loss of active ingredients on antimicrobial coverage; iii) the effect of probenecid co-administration to improve coverage; and iv) the characterization of high urinary amoxicillin exposure with consequences for use in urinary tract infections.

## INTRODUCTION

Amoxicillin is the most commonly used antibiotic globally (1, 2). It is a first-line antibiotic recommended for empirical treatment of many common infections listed within the World Health Organization (WHO) AWaRe classification, particularly community-acquired pneumonia (CAP) and ear, nose, and throat (ENT) infections (3). Despite this, there is a relatively limited understanding of the pharmacokinetics, pharmacodynamics, and clinical pharmacology of amoxicillin (4–6), with population pharmacokinetic (popPK) models having only recently been developed and only for orally administered (p.o.) amoxicillin (4, 7).

The most common and problematic pathogens of CAP and ENT infections are *Streptococcus pneumoniae* and *Haemophilus influenzae* (8, 9). While wild-type *S. pneumoniae* have relatively low amoxicillin minimum inhibitory concentrations (MICs) (with an epidemiological cutoff [ECOFF] value of 0.06 mg/L), mutations in penicillin-binding proteins (PBP) cause incremental decreases in ß-lactam susceptibility, producing a continuous distribution of amoxicillin MICs up to 16 mg/L (10), some of which can still be treated with amoxicillin. *H. influenzae* has a wild-type amoxicillin MIC distribution ranging up to 2 mg/L. However, *H. influenzae* can produce penicillinases and/or have PBP3 mutations conferring resistance, causing a bimodal distribution, with resistant isolates usually having MICs > 8 mg/L (9). Epidemiologically, ß-lactam resistance in both pathogens is a significant global problem, with regional resistance rates > 30% for both (11, 12). A PK/PD target for amoxicillin in invasive pneumococcal disease of the percentage of time the free fraction is above the MIC (*f*T >MIC) of 40% has been derived in a murine model (5), and clinical data suggest this is the target for both *S. pneumoniae* and *H. influenzae* (6)*. Streptococcus pyogenes* is another problematic target pathogen for amoxicillin, particularly in pharyngitis. However, these are universally penicillin-susceptible with low amoxicillin MICs (<0.125 mg/L) (10, 13), with adequacy of ß-lactam treatment options less of a concern.

Physiology-based pharmacokinetic (PBPK) modeling is an *in silico* modeling technique that uses information from human anatomy, physiology, and biochemistry, alongside insights into the pharmacological and physicochemical properties of a drug to predict the pharmacokinetics. Improvements in the understanding of human biology and computing power in recent years have dramatically improved the prediction accuracy of these models, with PBPK becoming a well-accepted component of regulatory filing and regimen selection (14–16). PBPK modeling has been used to model the effects of amoxicillin pharmacokinetics in special populations (i.e., pregnant and bariatric individuals) (17–19) or particular pharmacological scenarios (i.e., specific drug-drug interactions [DDIs] and novel formulations) (20–22), but the application of these has been limited to their specific contexts. More broadly, PBPK modeling of antibiotics has been used to assess antimicrobial PK/PD target attainment in specific populations and/or non-systemic sites of deposition, but not for amoxicillin (23–29). Here, we set out to construct a general PBPK model of amoxicillin, which aims to i) assess the compartmental pharmacokinetics and efficacy of amoxicillin to treat infections caused by *S. pneumoniae, H. influenzae,* and other pathogens; ii) understand the urinary exposures of amoxicillin; iii) assess the effects of decreased active compound or omitted doses on the adequacy of amoxicillin regimens; and iv) assess the potential of probenecid co-administration to improve amoxicillin exposure through inhibition of the Organic Anion Transporter 3 (OAT3) transporter.

## RESULTS

### Amoxicillin PBPK model development

A PBPK model was developed with the initial characteristics summarized in Table 1. This model was used to simulate the administration of i.v. 1 g amoxicillin as a bolus in a single simulated adult, compared to the available plasma and urinary PK data for this regimen (30–33). Iterative changes in distribution prediction models determined that the Rodgers and Rowland method of predicting partition coefficients (34) and the “PK-sim standard” method prediction of cellular permeabilities most accurately capture the predicted drug exposure profile of amoxicillin. Lipophilicity was refitted using the available observed data to generate an estimate of −0.21 LogMA, yielding drug distribution simulations that best matched the observed data. Fitted renal and hepatic clearance parameters of amoxicillin were 2.79 and 1.33 mL/h/kg, respectively.

**TABLE 1:** Initial characteristics of amoxicillin used to create the PBPK model

Property	Parameter value
Molecular weight	365.4
Lipophilicity (LogMA)	−2^*a*^
Fraction unbound^*b*^	0.83 (35)
pKA1/2	3.2/11.7 (36)
Solubility (g/L)	Solubility function by pH available (37)
Renal clearance (mL/h/kg)	1.905 (38)
Extra-renal clearance (mL/h/kg)	1.41 (38)

^*a*^ Prediction of LogP using XLogP3 via PubChem. In the absence of a LogMA value, a LogP value is a reasonable initial estimate in PK-Sim.

^*b*^ Specific protein binding targets unknown.

**Fig 1:**
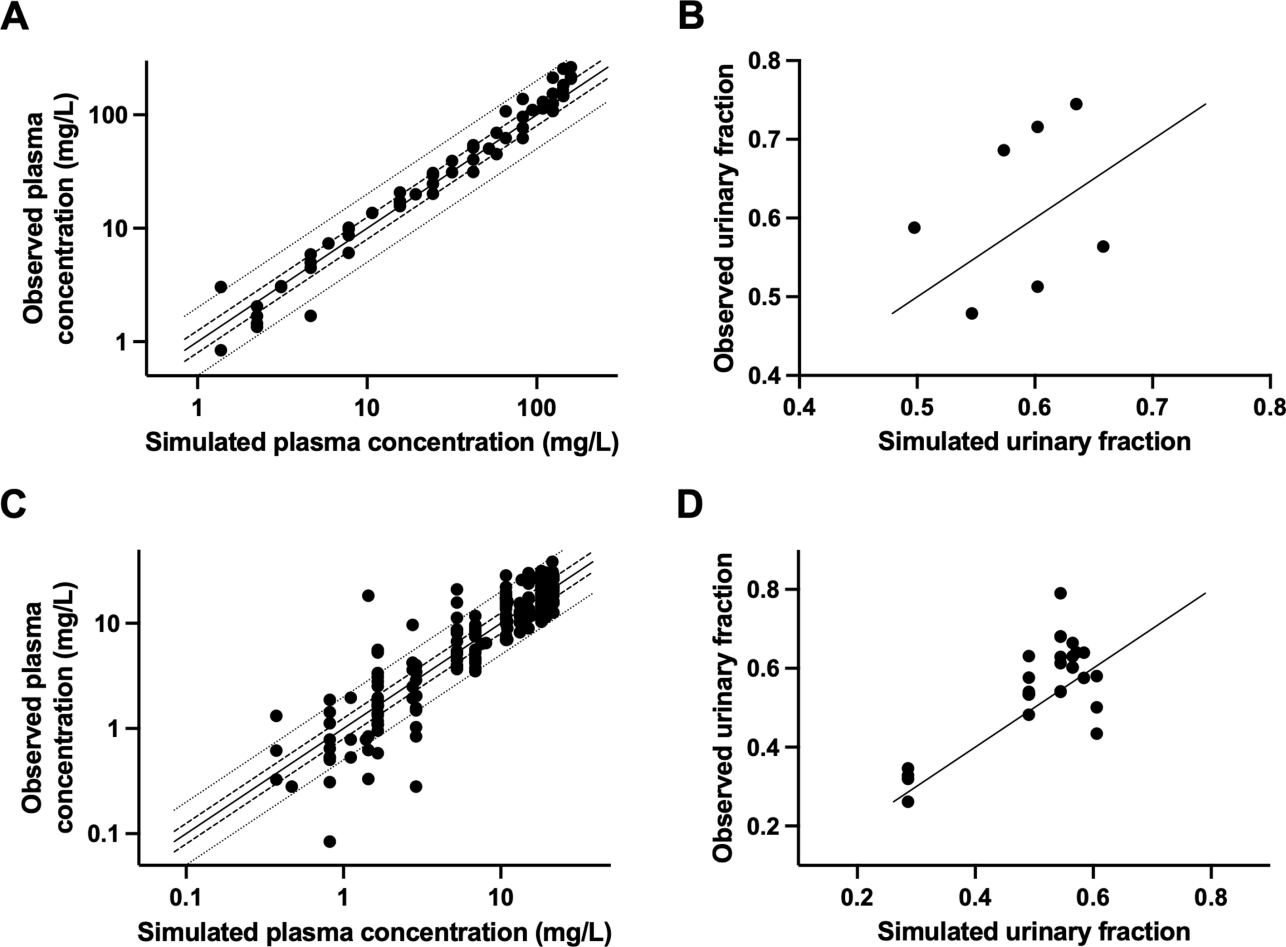
Observed vs predicted plots for simulated outputs of 1 g i.v. amoxicillin as a bolus (**A**: plasma concentration; **B:** urinary concentration) and 500 mg p.o. amoxicillin (**C**: plasma concentration; **D**: urinary concentration) vs observed data from multiple published data sets (30–33, 35, 38–40, 42–59). In all panels, the solid line represents the identity line. For panels **A and C**, the dashed and dotted lines depict the 1.25- and 2-fold deviation lines, respectively. Deviation lines are not provided for panels **B and D** due to the bounded nature of urinary fraction data.

**Fig 2:**
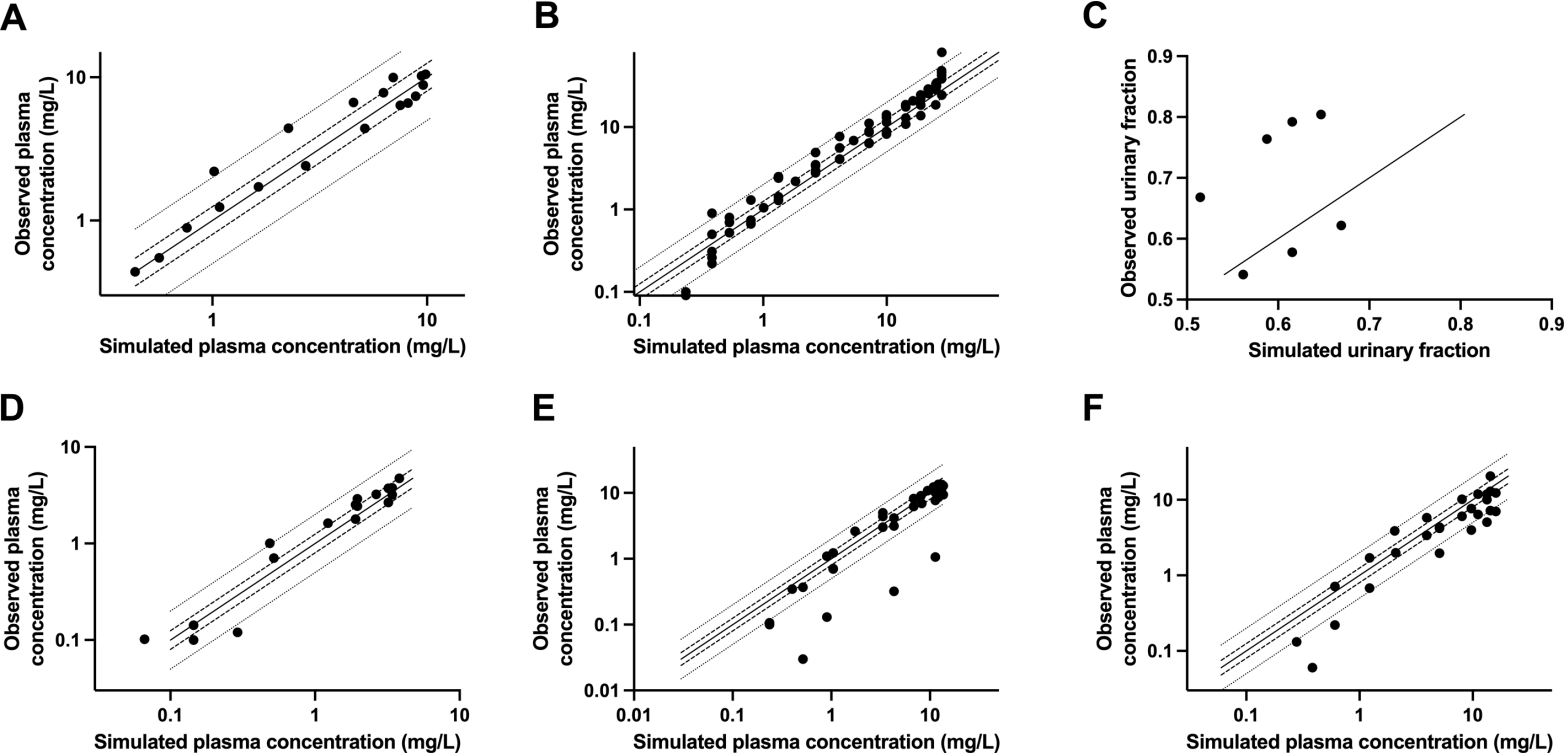
Observed vs predicted plots for final intravenous (**A–C**) and oral (**D–F**) PBPK models applied to non-training data sets compared to available observed data (30, 31, 35, 38–41, 52, 57, 60–63). Simulated regimens are 250 mg i.v. amoxicillin 33 min infusion (**A**), 500 mg i.v. amoxicillin bolus (**B and C**), p.o. 250 mg amoxicillin (**D**), p.o. 875 mg amoxicillin (**E**), and p.o. 1 g amoxicillin (**F**). Urinary data not shown for regimens with limited urinary fraction data, but these are detailed in Tables 2 and 3. In all panels, the solid line is the identity line. In plasma exposure panels, the dashed and dotted lines represent 1.25-fold and 2-fold error lines, respectively. Deviation lines are not provided for panel **C **due to the bounded nature of urinary fraction data.

This model performed satisfactorily with all i.v. regimens (30, 31, 38–41). Consequently, the effect of OAT3 was incorporated into the overall estimate of renal clearance. The available probenecid co-administration data suggest renal clearance at approximately the unmodified glomerular filtration rate (42). For additional clearance mediated via OAT3, a K_m_ of 282 μM was determined *in vitro* and used in the model with a fitted *in vivo* V_max_ of 50,422.43 pmol/min/pmol transporter. An empirical apical efflux transporter was added to reflect net onward transport into the urine, absent kinetic characterization for specific apical transporters. With a notional transporter concentration of 1 μmol/L, notional fitted K_m_ and V_max_ values of 1.53 mM and 383.9 pmol/min/pmol transporter, respectively, were determined, fitting the model to available urinary PK data. The final performance of this PBPK model, with simulations of 1 g i.v. bolus, 500 mg i.v. bolus, and 250 mg i.v. infusion (33 min), was satisfactory compared to available observed data (Fig. 1 and 2; Table 2). The PBPK model predictions for C_max_ were generally lower than many IV bolus data sets. This is due to the perfect mixing assumed *in silico,* which is not readily seen *in vivo* for bolus injections.

**TABLE 2:** Summary of the pharmacokinetic performance of simulations in comparison to training and validation data sets for i.v. amoxicillin regimens

Simulated regimen	Data set	Number of subjects in study	C_max_ (mg/L)	Fold error from simulation	AUC_0-24h_ (mg.h/L)	Fold error from simulation	End-study urinary fraction	Fold error from simulation
1 g amoxicillin bolus								
	Simulation		57.52		53.76			
	Hill *et al.*	7	93.72	1.6	57.15	1.1	0.75	1.17
	Mastrandrea *et al.*	10	74.1	1.3	41.56	0.8	0.56	0.85
	Staniforth *et al.*	8	90.84	1.6	58.96	1.1	–	–
	Westphal *et al.*	8	77.6	1.3	50.41	0.9	–	–
500 mg i.v. amoxicillin bolus								
	Simulation		28.30		25.99			
	Arancibia *et al.*	9	42.6	1.5	33.6	1.3	–	–
	Hampel *et al.*	10	24.4	0.9	19.38	0.7	–	–
	Hill *et al.^a^*	7	80.86	2.9	28.45	1.1	0.8	0.65
	Mastrandrea *et al.*	10	45.7	1.6	30.22	1.2	0.62	0.67
	Witkowski *et al.*	10	38.53	1.4	22.81	0.9	–	–
250 mg i.v. amoxicillin 33 min infusion								
	Simulation		10.04		12.77			
	Zarowny *et al*.	8	10.5	1.0	11.5	0.9	–	–
Weighted Geometric Mean fold error^*b*^				1.41		0.97		1.00

^*a*^ The C_max_ value for the 500 mg i.v. bolus in the study by Hill *et al.* appears erroneously high, with the concentration out of keeping with the expected linearity when compared to the value for 1 g i.v. bolus or the other 500 mg i.v. bolus regimens in the study by Hill *et al.* This is assumed due to experimental factors as appropriate linearity resumes in subsequent data points. This data point has been included regardless of the transparency.

^*b*^ Geometric means of fold errors weighted by the number of participants in each study. Each end-study urinary fraction is from a different time point for each data set. Fold errors are determined from the time-matched simulation urinary fraction. -, no urinary PK data available in the relevant study.

**TABLE 3:** Summary of pharmacokinetic performance of simulations in comparison to training and validation data sets for orally administered amoxicillin regimens^*a*^

Simulated regimen	Data set	Number of subjects in study	C_max_ (mg/L)	Fold error from simulation	AUC_0-24h_ (mg.h/L)	Fold error from simulation	End-study urinary fraction	Fold error from simulation
500mg p.o. amoxicillin								
	Simulation		7.71		22.83			
	Adam *et al.*	12	6.61	0.9	18.25	0.8	0.68	1.19
	Arancibia *et al.*	9	9.5	1.2	26.85	1.2	–	–
	Desager *et al.*	16	4.51	0.6	12.54	0.5	–	–
	Foroutan *et al.*	12	8.5	1.1	30.66	1.3	–	–
	Gordon *et al.*	8	7.61	1.0	21.56	0.9	0.6	1.05
	Guibert *et al.*	6	7.9	1.0	28.1	1.2	–	–
	Hampel *et al.*	10	5.78	0.7	15.94	0.7	–	–
	Hoizey *et al.*	3	13.71	1.8	38.85	1.7	–	–
	Lode *et al.*	13	5.98	0.8	19.73	0.9	0.58	1.00
	Modr *et al.*	10	10.72	1.4	44.04	1.9	–	–
	Morasso *et al.*	8	5.59	0.7	15.73	0.7	–	–
	Motta *et al.*	12	7.72	1.0	28.42	1.2	–	–
	Neu	8	9.34	1.2	27.85	1.2	0.79	1.39
	Sourgens *et al.*	48	5.62	0.7	15.81	0.7	–	–
	Staniforth *et al.*	18	8.03	1.0	21.73	1.0	–	–
	Sum *et al.*	6	7.3	0.9	21.81	1.0	0.64	1.10
	Sutherland *et al.*	12	10.8	1.4	29.68	1.3	0.68	1.19
	Verbist	10	7.87	1.0	15.88	0.7	0.64	1.12
	Vree *et al.*	36	7.87	1.0	21.28	0.9	–	–
	Wise *et al.*	6	9.4	1.2	27.93	1.2	0.66	1.15
	Witkowski *et al.*	10	5.54	0.7	17.72	0.8	–	–
	Zhang *et al.*	12	7.63	1.0	26.5	1.2	–	–
1g p.o. amoxicillin								
	Simulation		14.55		45.07			
	Paulsen *et al.*	12	7.22	0.5	24.94	0.5	–	–
	Sutherland *et al.*	22	20.6	1.3	54.147	1.2	–	–
	Zhang *et al.*	12	12.94	0.8	50.53	1.1	–	–
875mg p.o. amoxicillin								
	Simulation		13.61		40.35			
	Burkhardt *et al.*	12	9.4	0.7	27.93	0.7	0.54	0.9
	Ciric *et al.*	24	13.51	1.0	41.67	1.0	–	–
	Fraschini *et al.*	10	11.23	0.8	30.35	0.8	0.51	0.88
250mg p.o. amoxicillin								
	Simulation		3.83		11.34			
	Motta *et al.*	18	3.2	0.8	9.58	0.8	–	–
	Zhang *et al.*	12	4.74	1.2	14.88	1.3	–	–
Weighted Geometric Mean fold error^*a*^				1.0		1.0		1.08

^*a*^ Geometric means of fold errors weighted by number of participants in each study. Each end-study urinary fraction is from a different time point for each data set. Fold-errors are determined from the time-matched simulation urinary fraction. -, no urinary PK data available in the relevant study.

All non-absorption-related parameters were fixed for this amoxicillin PBPK model and used to develop a p.o. model. Initially, a simulation of p.o. 500 mg amoxicillin was used compared to the available PK data (35, 38–40, 42–59) to identify oral absorption parameters. An additional PK data set was available (64) but was omitted from the analysis due to uncertainty over the number of individuals it was from and risk that data may be duplicated in other papers. Amoxicillin is a known substrate for the mucosal peptide transporter 1 (PEPT1) (65) and, potentially, a mucosal OATP transporter (66), which likely explains the observed saturable absorption of amoxicillin (7). However, the current version of PK-Sim could not mechanistically reproduce the known saturable transporters of amoxicillin with sufficient fidelity to accurately simulate high-dose p.o. exposure. Therefore, an empirical transcellular permeability parameter was used to incorporate both passive and active absorption routes. Parameters of dissolution shape and time (50% dissolved) with a Weibull dissolution model and total intestinal transcellular permeability were therefore co-fitted to the observed data for p.o. 500 mg amoxicillin, giving values of 1.44 for dissolution shape, 59.73 min for 50% dissolution time, and 2.09 × 10^−3^ cm/min for intestinal transcellular permeability. These estimates for the absorption parameters predicted plasma and urine PK satisfactorily against the observed data for a p.o. 500 mg regimen (Fig. 1). This was validated against data for p.o. 1 g, 875 mg, and 250 mg regimens for which there were sufficient PK data (35, 52, 57, 60–63), with satisfactory performance (Fig. 2 and Table 3). The model performed poorly against PK data sets with a p.o. 3 g regimen of amoxicillin (52, 60, 67, 68), with significant systematic overestimation of the predicted plasma concentrations. This is likely due to the saturability of amoxicillin absorption mechanisms (7), which were not properly configured within the final PBPK model. Therefore, the model is not suitable for modeling doses above p.o. 1 g.

### Simulations

**Fig 3:**
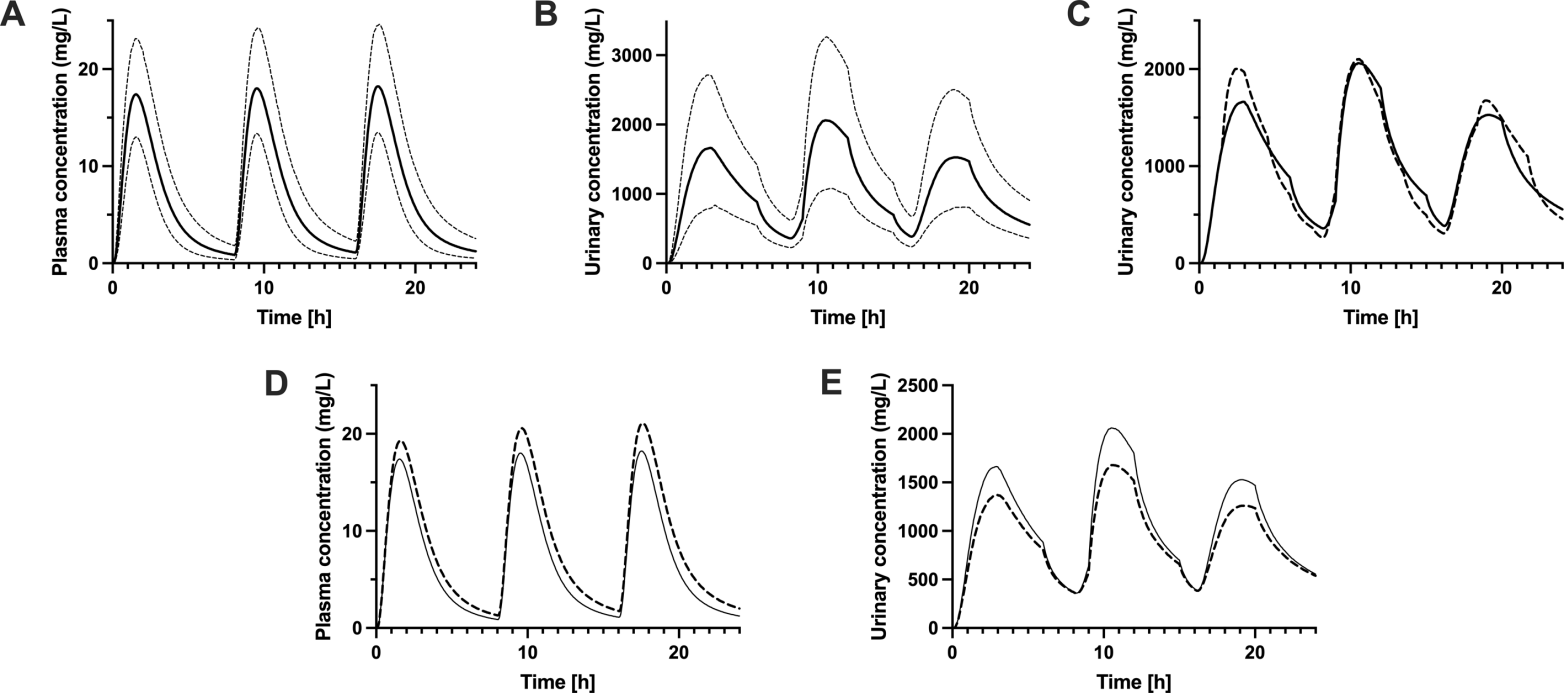
Simulation output of p.o. 1g amoxicillin q8h in a population of 1,000 adults. Panels **A and B**: simulated plasma (**A**) and urine (**B**) median concentrations (solid line) and 2.5%/97.5% centiles (dashed lines); Panel **C**: comparison of the median urinary concentrations with a healthy micturition frequency (solid line) and a doubled micturition frequency (dashed line) during receipt of p.o. 1 g amoxicillin q8h; Panels **D and E**: comparison of median plasma (**D**) and urinary (**E**) concentrations with (dashed line) and without (solid line) simulated co-administration with p.o. 500 mg probenecid q8h.

Simulations of plasma and urine amoxicillin exposures were performed in a simulated European adult population of 1,000 individuals for the following regimens: p.o. amoxicillin 500 mg and 1 g q8h, and i.v. amoxicillin 1 g and 2 g (q8h and q6h) given as a 30 min infusion (69). Co-administration of p.o. amoxicillin 1 g q8h with p.o. probenecid 500 mg q8h was also conducted. Exemplar plasma and urinary time-concentration profiles from the simulation of regimen p.o. 1 g amoxicillin q8h and the effects of co-administration with probenecid are shown in Fig. 3.

**Fig 4:**
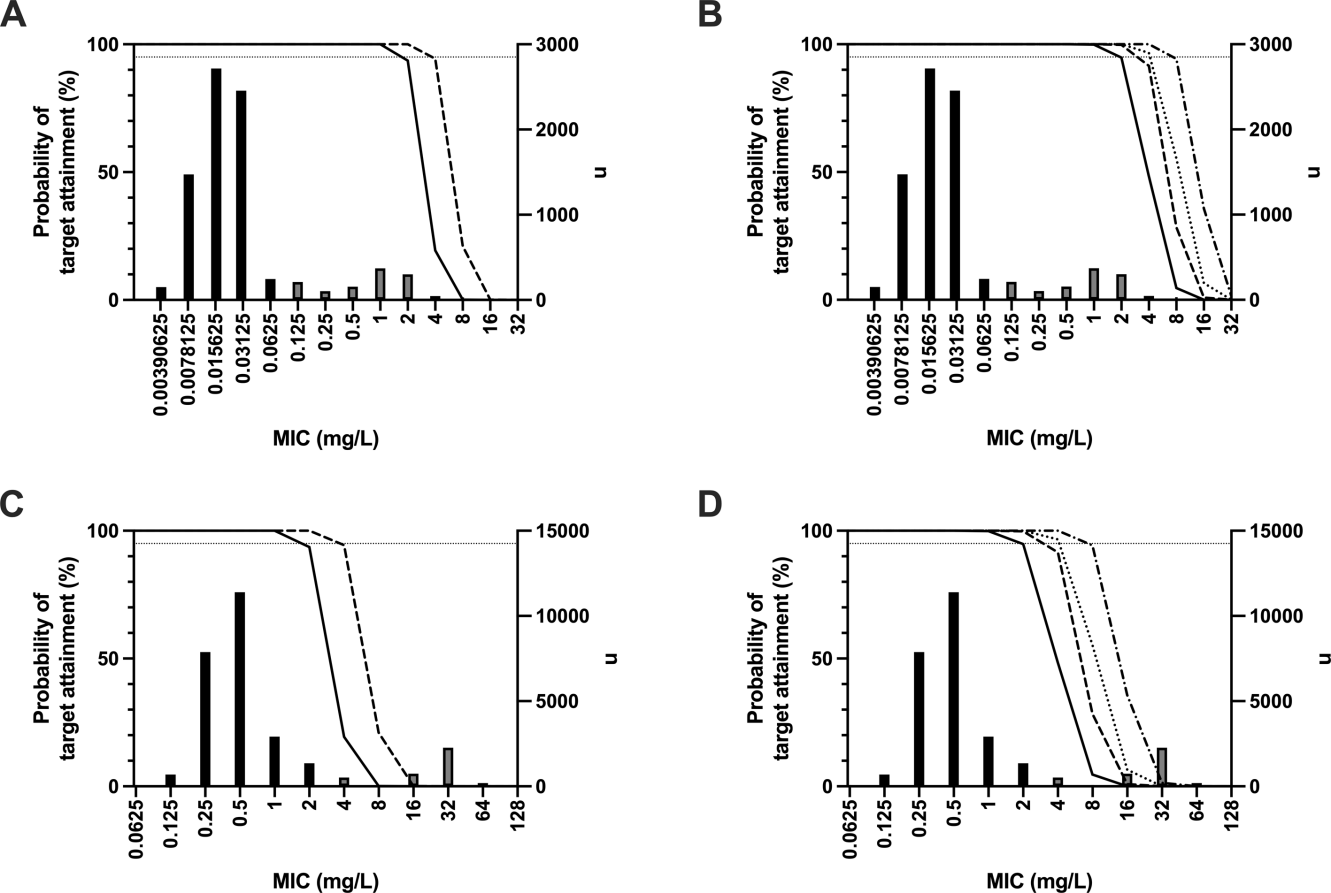
PTA analyses for oral (panels **A and C**) and intravenous (**B and D**) amoxicillin regimens for an *f*T > MIC target of 40% with respect to the amoxicillin MIC distributions of *S. pneumoniae* (**A and B**) and *H. influenzae* (**C and D**) (10). Black bars indicate the wild-type distribution of each species up to the ECOFF. Gray bars indicate non-wild-type strains with resistance mechanisms. In panels **A and C**: solid line = p.o. 500 mg amoxicillin q8h; dashed line = p.o. 1 g amoxicillin q8h. In panels **B and D**, solid line = i .v. 1 g amoxicillin q8h; dashed line = i .v. 1 g amoxicillin q6h; dotted line = i .v. 2 g amoxicillin q8h; dot-dashed line = i .v. 2 g amoxicillin q6h. All i.v. regimens were given with a 30 min infusion. The horizontal dotted line indicates 95% target attainment.

Using the *f*T>MIC PK/PD target of 40% for *S. pneumoniae* and *H. influenzae* (5, 6). the probability of target attainment (PTA) analyses were conducted for p.o. and i.v. amoxicillin regimens (Fig. 4A and B). Here, both oral regimens clearly achieved >95% target attainment for susceptible and relatively resistant *S. pneumoniae* isolates up to amoxicillin MICs of 1 mg/L and 2 mg/L for 500 mg and 1 g p.o. amoxicillin regimens, respectively. Correspondingly, only the p.o. 1 g amoxicillin regimen could achieve >95% target attainment for the *H. influenzae* ECOFF of 2 mg/L. Almost all i.v. regimens achieved >95% PTA at 2 mg/L (1 g q8h achieved 94.8% PTA at 2 mg/L). Both 2 g i.v. amoxicillin regimens (q8h and q6h) also managed to achieve >95% PTA at 4 mg/L.

**Fig 5:**
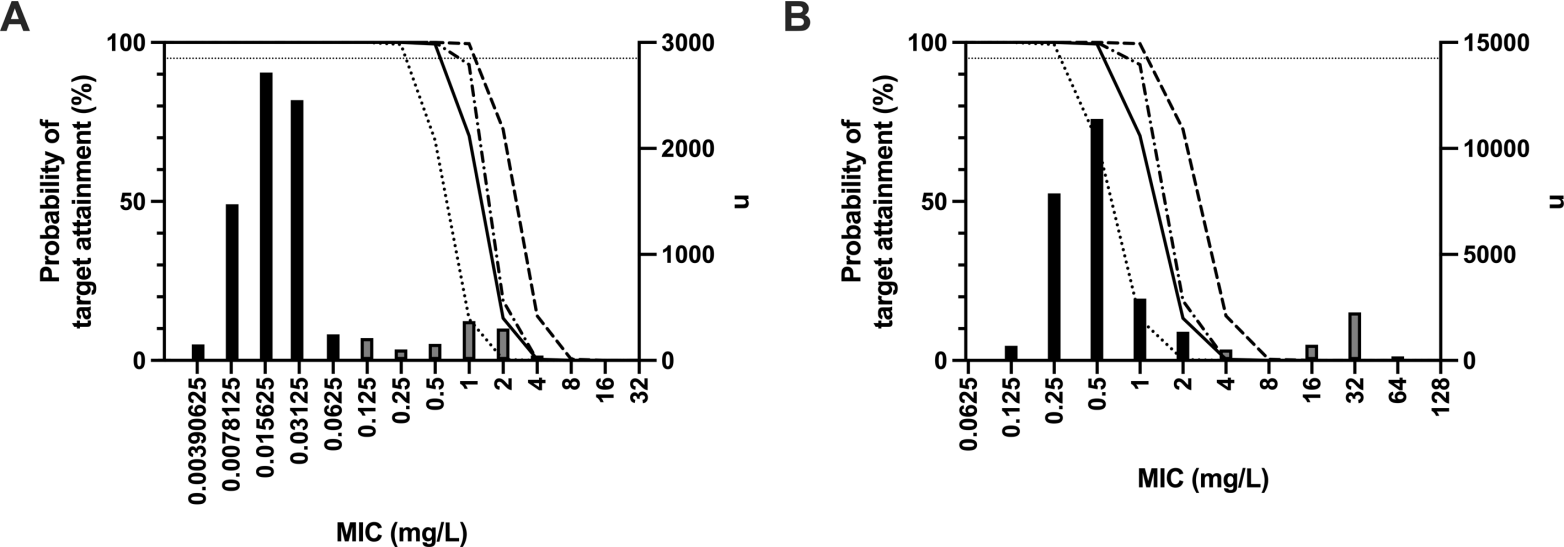
PTA analyses for a range of conditions potentially complicating oral amoxicillin regimens in LMIC settings for an *f*T > MIC target of 40% with respect to the amoxicillin MIC distributions of *S. pneumoniae* (**A**) and *H. influenzae* (**B**) (10). Black bars indicate the wild-type distribution of each species up to the ECOFF. Gray bars indicate non-wild-type strains with resistance mechanisms. Solid and dashed lines indicate p.o. 500 mg and 1 g amoxicillin q8h regimens, respectively, with one dose omitted in a 24 h period. The dot-dashed line indicates a p.o. 500 mg amoxicillin q8h regimen with 50% active ingredients. The dotted line indicates the same, but with an omitted dose in a 24 h period.

To determine the effects of counterfeit or degraded amoxicillin and non-compliance, three scenarios were simulated (Fig. 5). First, a 50% reduction in the active material, to illustrate an upper-end reduction due to counterfeit or degradation caused by improper storage, as mentioned in the published literature (70, 71), which reduced the MIC at which 500 mg p.o. amoxicillin q8h achieved a 95% PTA by one 2-fold dilution. Second, an omission of one dose in 24 h reflecting partial non-compliance was simulated, reducing the MICs at which >95% PTA was achieved to 0.5 mg/L and 1 mg/L for 500 mg and 1 g p.o. amoxicillin regimens, respectively. Finally, a combination of the two conditions was co-simulated, reducing the MIC at which >95% PTA was achieved further to 0.25 mg/L and 0.5 mg/L for the same regimens.

The addition of probenecid to p.o. amoxicillin 1 g q8h only marginally changed the drug exposure in comparison to amoxicillin monotherapy (Fig. 3D). However, this increased the MIC at which > 95% PTA was achieved to 4 mg/L. Probenecid co-administration did reduce the peak urinary concentration (Fig. 3E), but overall urinary exposure remained high.

Specific amoxicillin PK/PD targets have not been determined for relevant urinary pathogens (4), so only a descriptive analysis was performed on amoxicillin urinary exposure. As shown in Fig. 3, urinary exposures of amoxicillin were considerable, even with low doses of amoxicillin. More frequent micturition did not fundamentally change this (Fig. 3C). For p.o. 500 mg amoxicillin q8h, this meant that amoxicillin concentrations were >32 mg/L for 95% of the time for all 1,000 simulated individuals.

## DISCUSSION

Here, we have developed the first broadly generalizable PBPK model of amoxicillin intended for integrated plasma and urinary exposure analyses across multiple dosing contexts. This enables a deeper understanding of the use of amoxicillin in three ways. First, this PBPK model allows an alternative model for predicting the population exposures of antibiotics where popPK models are not available, as is the case for many EML drugs (4), and available published time-concentration data are insufficient to construct one. While amoxicillin popPK models exist for oral regimens (7, 72), popPK models for intravenous regimens are lacking. This PBPK model provides a population-level prediction of amoxicillin exposure for intravenous regimens in the absence of such a popPK model, allowing assessment of the adequacy of these regimens.

Second, this approach attempts to understand urinary amoxicillin exposures in a way typically overlooked by standard popPK analyses, due to the practical difficulty of capturing urinary time-concentration profiles in PK studies and modeling physiological urine accumulation and micturition in popPK models. This approach allows insights into the adequacy of site-specific infections (i.e., UTIs) and informs other lines of research (73). There is also the potential to use PBPK models like this to predict the best estimates of drug exposures in tissues not amenable to sampling, where they can be used as a best approximation of antibiotic exposure in compartments where empirical data are difficult or impossible to obtain (74). Finally, PBPK models allow for prediction of drug-drug interactions (DDIs) (74), as we do here with probenecid co-administration.

For invasive pneumococcal disease, our model predicts that all tested regimens achieve >95% attainment for the wild-type distribution of *S. pneumoniae* and relatively resistant phenotypes with amoxicillin MICs up to 1 mg/L (Fig. 4), even for the lowest-dose regimens, extending to 2 mg/L for 1 g p.o. amoxicillin q8h and 4 mg/L for 2 g i.v. amoxicillin q8h and q6h. The same rates of attainment apply to *H. influenzae*. Here, the model output confirms the need for an increased dose of amoxicillin to treat wild-type *H. influenzae*, in keeping with guideline recommendations (75).

However, certain contexts can hinder the adequacy of amoxicillin for treating these pathogens. Counterfeit medication is an issue for amoxicillin in low- and middle-income countries (LMICs), with amoxicillin active ingredient rates as low as 59% being reported (70). Additionally, hot and humid conditions in tropical and subtropical countries can lead to ß-lactam ring degradation, with amoxicillin degradation rates as high as 41% being reported (71). This is further compounded by frequent antibiotic non-adherence in LMIC settings (76, 77). Our simulations of amoxicillin with 50% active ingredients (reflecting counterfeit drugs or degradation) and relative non-compliance show that while p.o. amoxicillin regimens are still adequate for wild-type *S. pneumoniae*, adequacy against relatively resistant isolates and susceptible *H. influenzae* strains declines to varying degrees. This potentially compromises successful treatment in settings where infections with these latter organisms are prevalent and microbiological identification capability is not readily available.

In this work, we also explored the possible benefits of using probenecid to enhance amoxicillin exposure. This has been a strategy that has recently attracted interest as a means to improve drug exposures of ß-lactam antibiotics (78). The improvements to amoxicillin exposure with probenecid co-administration predicted by this PBPK model are marginal (Fig. 3C and D). However, it did increase the amoxicillin MIC at which the *S. pneumoniae* target is attained by 2-fold, raising this as a potential modality to treat relatively resistant *S. pneumoniae* isolates with MICs up to 4 mg/L. Other OAT3 inhibitors may have different effects depending on their OAT3 inhibitory kinetics, but these require construction of specific PBPK models in order to allow individual assessment of the DDIs with these molecules.

The other main application for our model is the treatment of urinary tract infections (UTIs). UTIs were historically an indication for amoxicillin in the AWaRe handbook, but this indication was removed due to rising amoxicillin resistance in UTI pathogens globally (79). However, it remains recommended in many high-income guidelines where relevant susceptibility data are available (80). The high urinary amoxicillin exposure predicted by our PBPK model (Fig. 3) provides further insights here. The PK/PD target for amoxicillin for enterococci or Enterobacterales has not been characterized, nor for urinary conditions in general. However, our model predicts urinary concentrations of >32 mg/L for almost all of the dosing period for p.o. 500 mg amoxicillin q8h. This corroborates the recommendation to use amoxicillin for UTIs caused by susceptible Enterobacterales (75). However, it may also explain the apparent success of p.o. amoxicillin to treat UTIs caused by *Enterococcus faecium* (81–83)—an organism typically considered intrinsically resistant to amoxicillin with a wild-type amoxicillin MIC distribution up to 32 mg/L (10).

This study has limitations in its application of PBPK modeling. First, amoxicillin pharmacokinetics likely involve multiple transporters and enzymes. However, the model incorporated only the most strongly implicated transporter, OAT3. As a result, we relied on empirical parameterization of hepatic metabolism and non-OAT3-mediated transport. An implication of this is that the saturable absorption of amoxicillin (7) could not be modeled above doses of 1 g administered orally. While 3 g PO doses are not commonly used today, they have been used for the treatment of UTIs and prophylaxis for endocarditis during dental procedures (84, 85), and our model cannot inform these particular regimens. Determination and incorporation of relative enzyme and transporter kinetics may further improve the model, particularly for predicting wider-ranging DDIs and incorporating the saturable absorption into the model.

Second, there are some limitations to the urinary concentration predictions. We have validated the predictions of the amount of amoxicillin renally excreted in our model, but the performance is less precise than for plasma compared to observed data (Tables 2 and 3), acknowledging that urinary concentration data from pharmacokinetic studies have a significant degree of measurement uncertainty (86). There is a lack of a validated mechanistic kidney model in PK-Sim to dynamically simulate physiological urine production; however, we simulated a fixed urine production of 1 mL/kg/h in order to predict the urinary concentration of amoxicillin. Physiological urine production rates are contingent on a variety of factors not simulated in our model, and our urine concentration simulation data should be interpreted accordingly.

Finally, although we used this model to make inferences relevant to LMIC settings, PK-Sim does not have validated population models for non-high-income countries. As such, all simulations were conducted on a simulated population derived from European (Caucasian) physiological parameters. This is a known issue with PBPK modeling (87), and further data are needed to accurately simulate populations relevant to LMICs. Further, our analysis and conclusions are limited to systemic and urinary exposure. A PBPK model predicting local compartmental drug exposure in, e.g., tonsillar tissue, lung epithelial lining fluid (ELF), and middle ear fluid, may give further insights into the adequacy of regimens for infections confined to these compartments. Unfortunately, amoxicillin ELF data are limited to a time-delinked study (88), and PBPK representations of tonsillar/middle ear anatomical compartments have not been constructed and validated for PK-Sim. Finally, our PTA analyses use MIC data from the EUCAST database and are not intended to be epidemiologically representative for a global context.

However, despite these limitations, we have constructed a high-performing PBPK model of amoxicillin (Fig. 1 and 2, Tables 1 and 2) and applied this model to multiple applications. In particular, we have i) provided insights into the PK/PD coverage of intravenous and orally administered amoxicillin for respiratory and ENT infections caused by *S. pneumoniae* and *H. influenzae*; ii) explored how non-compliance and active ingredient loss can impair the adequacy of amoxicillin for these indications; iii) demonstrated the likely effects of probenecid co-administration in boosting the adequacy of amoxicillin; and iv) described the likely urinary pharmacokinetics in a real-life context, with the impact on use of amoxicillin for the treatment of UTIs of various etiologies.

## MATERIALS AND METHODS

### Pharmacokinetic data extraction

Pharmacokinetic data for amoxicillin were sourced via a systematic review of the literature. A search with the term “amoxicillin pharmacokinetics” was conducted in MEDLINE without date or language restriction. All abstracts were screened for relevance, with full text for relevant abstracts reviewed. Papers detailing plasma +/− urine time-concentration data for amoxicillin in intravenously or orally administered forms in healthy volunteers in a standard pharmacokinetic (PK) study setting (i.e., not studies examining interactions, food effects, or enhanced formulations) were selected, with PK data extracted. Where precise time-concentration values were not given, values were extracted from individual figures with WebPlotDigitizer (https://automeris.io/WebPlotDigitizer/).

### OAT3 transporter kinetics

Following the likely association of amoxicillin with the OAT3 SLC transporter (28), OAT3 transporter kinetics for amoxicillin were determined by Cyprotex Discovery Ltd. (UK). HEK293 cell lines stably expressing either an OAT3 transporter or a non-transporter expressing control were seeded at approximately 3–4 × 10^5^ cells per well in 24-well poly-D-lysine plates to achieve a pre-assay confluence of 80%–95%. These cells were incubated at 37°C in 5% CO_2_ for 24 h in a humidified incubator. The cells were then washed twice with pre-warmed uptake buffer (HBSS containing 10 mM HEPES, pH 7.4) and then pre-incubated with 400 μL warm uptake buffer for 15 min. Following this, the uptake buffer was removed, and amoxicillin dissolved in DMSO was added to the wells and incubated at 37°C for 2 min. Seven different concentrations of amoxicillin were tested (1, 2, 5, 10, 25, 100, and 500 μM) in triplicate.

Reactions were then terminated with removal of the test solution, immediate washing of cells twice with ice-cold uptake buffer, and placing the plates on ice. Cells were lysed, and amoxicillin concentrations in the samples were quantified by LC-MS/MS. Samples of the lysate were also taken to determine the protein concentration in each well with a bicinchoninic acid (BCA) assay (Pierce BCA protein assay, Thermo Fisher Scientific, USA).

Uptake into control cells was subtracted from that into the transporter-containing cells to determine transporter-mediated uptake. This was plotted versus nominal substrate concentrations to determine K_m_/V_max_ parameters using GraphPad Prism (GraphPad Software, USA).

### PBPK model development

An amoxicillin PBPK model was constructed with the open-source software PK-Sim v12.2 (Open Systems Pharmacology). Initial physicochemical properties were sourced from targeted literature searches for specific properties. Initial pharmacological parameter estimates (e.g., renal clearance) were sourced from parameters calculated from published amoxicillin PK studies, with an assumption that initial parameter estimate precision could be improved during model development. In the absence of mechanistic characterization of amoxicillin metabolism, all metabolism was attributed to hepatic metabolism and parameterized as total hepatic clearance in the model. A simulated adult European male with median physiological parameters derived from the International Commission on Radiological Protection (ICRP) data set (89) served as the basis for simulations in model construction.

An initial PBPK model was developed using an i.v. administered regimen. Simulations of the regimen were used to improve the PBPK model predictions in two ways. First, different drug distribution prediction methodologies were iteratively used to determine which distribution model best fitted the observed data. Second, parameter values of lipophilicity and clearance (renal and hepatic) were improved via parameter estimation using the Levenberg-Marquardt algorithm against the available observed plasma and urine time-concentration data. Observed data sets were weighted according to the number of participants in each study.

Once final estimates of renal clearance were determined, total renal clearance was mechanistically divided into glomerular filtration at the physiological glomerular filtration rate (GFR) and active transport via OAT3 transporters, which mediate the influx of amoxicillin into renal interstitial cells, using the enzyme kinetic parameters determined from the *in vitro* study. As the experimental V_max_ could not be extrapolated to the *in vivo* context in the PBPK model, this parameter was optimized via fitting to the observed data by the same method as given above. Renal OAT3 expression was determined from relative expression from RT-PCR data and absolute expression levels sourced from the literature (90, 91). Non-renal expression of OAT3 was set to 0 to prevent non-physiological accumulation of amoxicillin in tissues where no onward transporters have been characterized. A variety of undetermined mechanisms of apical transport from the intracellular space into the urine and active reabsorption from the urine back into the intracellular space are likely to be present (65). Hence, an empiric apical transporter (“apical efflux transporter”) was incorporated at a notional concentration of 1 μmol/L. Notional K_m_/V_max_ parameters were then fitted to the plasma/urinary time-concentration data, assuming glomerular filtration and OAT3 transport were the same as determined above.

Once the simulated output of the PBPK model satisfactorily performed compared to the observed data, the performance of the model was assessed using a holdout subset of observed time-concentration data for different i.v. regimens. Once the PBPK model satisfactorily performed for all intravenous regimens, all parameters relevant to intravenous administration were fixed. A model for oral administration (p.o.) was then developed. Parameter estimates for intestinal permeability and dissolution kinetics of the formulation in the gut were fitted to the available PK data using the same approach to parameter identification as described above using the subset of PK data for a p.o. 500 mg regimen available from the literature search. The performance of the model was validated using the available hold-out PK data for other p.o. regimens, as described above.

### Population simulation

Simulations of clinically relevant regimens of amoxicillin were conducted in simulated population cohorts of 1,000 European adults aged 16–65 using the physiological parameters defined by the ICRP demographics (89). As the base PK-sim program simulates renally excreted amounts in the urine only, and not urine production, urine production was simulated using the OSPsuite R package using the simulation data exported from the base PK-sim program. Here, urine production of 1 mL/kg/h, as a typical healthy urine output value (92), was simulated using individual body weights, with an initial bladder urine volume of 50 mL and the simulated renally excreted amounts added dynamically to the model to produce a urinary drug concentration. Complete emptying of the bladder (i.e., urination) was simulated by reducing the urine volume to a 50 mL residual volume (93, 94) with the prevailing concentration. Simulated micturition occurred at 3, 6, 9, 12, 15, and 20 h from the start of the simulation based on the reported modal frequency of urinary micturition (95, 96). An alternative scenario simulating increased urinary frequency due to UTIs was conducted with micturition at 1.5, 3, 4.5, 6, 7.5, 9, 10.5, 12, 13.5, 15, 18.33, and 21.66 h from the start of the simulation based on the relative increase in micturition from the available data (97).

Population time-concentration data in plasma were used to determine the probability of target attainment for a target of 40%*f*T>MIC for *S. pneumoniae* and *H. influenzae*. Descriptive analysis of urine exposures was also conducted, without reference to a PK/PD target, given the absence of PK/PD characterization for relevant pathogens.

To determine if OAT3-mediated drug-drug interactions (DDIs) could be leveraged to improve amoxicillin exposure, a previously published PK-Sim model of probenecid (an OAT3 inhibitor) was used to simulate the PK effects of co-administration of amoxicillin and probenecid (98). Additional simulations were conducted to predict the effects of non-compliance with standard amoxicillin p.o. regimens and below expected active ingredient amounts, reflecting poorly stored or counterfeit medication.

## Data Availability

A copy of the final amoxicillin PBPK model file can be accessed at https://github.com/CDarlow/Amoxicillin-PBPK-model/. The open-source software required to use this model can be found at https://www.open-systems-pharmacology.org/.
